# Main Applications of Cyclodextrins in the Food Industry as the Compounds of Choice to Form Host–Guest Complexes

**DOI:** 10.3390/ijms22031339

**Published:** 2021-01-29

**Authors:** Antía Gonzalez Pereira, Maria Carpena, Paula García Oliveira, Juan Carlos Mejuto, Miguel Angel Prieto, Jesus Simal Gandara

**Affiliations:** 1 Nutrition and Bromatology Group, Analytical and Food Chemistry Department, Faculty of Food Science and Technology, University of Vigo, Ourense Campus, E32004 Ourense, Spain; antia.gonzalez.pereira@uvigo.es (A.G.P.); maria.carpena.rodriguez@uvigo.es (M.C.); paula.garcia.oliveira@uvigo.es (P.G.O.); 2 Centro de Investigação de Montanha (CIMO), Instituto Politécnico de Bragança, Campus de Santa Apolonia, 5300-253 Bragança, Portugal; 3 Department of Physical Chemistry, Faculty of Science, University of Vigo, Ourense Campus, E32004 Ourense, Spain; xmejuto@uvigo.es

**Keywords:** cyclodextrins, complexes, inclusion, applications, bioactive compounds, food

## Abstract

Cyclodextrins (CDs) are cyclic oligomers broadly used in food manufacturing as food additives for different purposes, e.g., to improve sensorial qualities, shelf life, and sequestration of components. In this review, the latest advancements of their applications along with the characteristics of the uses of the different CDs (α, β, γ and their derivatives) were reviewed. Their beneficial effects can be achieved by mixing small amounts of CDs with the target material to be stabilized. Essentially, they have the capacity to form stable inclusion complexes with sensitive lipophilic nutrients and constituents of flavor and taste. Their toxicity has been also studied, showing that CDs are innocuous in oral administration. A review of the current legislation was also carried out, showing a general trend towards a wider acceptance of CDs as food additives. Suitable and cost-effective procedures for the manufacture of CDs have progressed, and nowadays it is possible to obtain realistic prices and used them in foods. Therefore, CDs have a promising future due to consumer demand for healthy and functional products.

## 1. Introduction

Numerous investigations have been focused on developing relatively simple organic compounds able to catalyze organic reactions, like enzymes. Since the discovery of enzymes’ active sites, new models of synthesis of nonpeptide organic systems that could simulate the enzymatic behavior have been developed. Chemists have been developing more sophisticated molecular structures within the nanometric order. However, even though apparently the easiest way to solve it would be to assemble individual molecules directly, the most efficient alternative is to generate molecules with a complementary form capable of spontaneously self-organizing, resulting in orderly assemblies. This can be achieved, for example, with host and guest molecules [1].

Host–guest complexes are molecular aggregates stabilized via noncovalent bonds (for example, van der Waals, hydrogen bonds, and hydrophobic interactions), but never by complete covalent bonds. Host molecules are characterized by having an inner cavity where another molecule can be incorporated, this is, the guest molecule. Therefore, hosts will act as receptors and guests as substrates, inhibitors, or cofactors [2]. The resulting molecular inclusion complex can easily break under determined physiological environments [3].

Therefore, the establishment of these systems can improve physicochemical properties of the guest molecule. Different types of host molecules have been developed, but they are all characterized by acting as non-natural receptors capable of partial or total enclosure of the compounds of interest (drugs, active compounds, etc.). Likewise, numerous hosts-guest complexes have already been synthesized. Among them, crown ethers, cryptands, spherands, carcerands, and cyclodextrins (CDs) can be highlighted.

CDs are a group of relatively recent discovered compounds, which are also identified as cycloamyloses, cyclomaltoses, and Schardinger dextrins [4]. Their first reference dates from 1891. In this year, a crystalline product was isolated during a starch fermentation carried out by *Bacillus amylobacter*. Some authors reflected that this production was probably due to impurities in cultures and, consequently, it was supposed that CDs might be produced by cross-contamination with another species (*B. macerans*). Later, in 1903, two crystalline components were isolated: dextrins A and B, which were characterized by their lack of reducing power. Even so, the bacterial strain responsible of its production was unfortunately not preserved [5]. A year later, the same authors were able to isolate an organism capable of producing acetone and ethyl alcohol from plant materials that contained sugar and starch. In 1911, it was discovered that a strain of *B. macerans* produced large amounts of crystalline dextrins (25–30%) from starch too. These resulting products were named as crystallized dextrin α and crystallized dextrin β [4]. It was not until 1935 that the third type of dextrin (dextrin γ) was isolated. Several fractionation processes were also developed in order to produce CDs [5]. At that point, the structure of said molecules was still a mystery. It was not fully discovered until 1942, when the structure of two CDs (α and β) was established through X-ray crystallography. A few years later (1948), the same method was applied to γ-CDs. Later, in 1961, more types of CDs (9–12 residues) were discovered [6].

CDs are one of the most used hosts-guest complexes for organic molecules. Their structure consists of a cyclic arrangement of D (+)-glucopyranose units joined by α-(1 → 4) glycosidic links. They can be differentiated according to their number of glucoses in α-, β- and γ-CDs (6, 7, and 8 glucose units, respectively) (Table 1) [7,8]. Regarding its form, it is a truncated cone characterized as bearing primary and secondary OH groups, respectively, in the slim and wide rim. It is characterized by having cavities with hydrophobic properties. In this case, as in other complexes, Van der Waals and hydrophobic forces are responsible for keeping guest and host together with a partial or total adjustment of the cavity. Due to the establishment of an inclusion complex, guest reactivity can vary, making possible its use in a wide variety of fields [9]. Furthermore, as these receptors can improve bioavailability, they are suitable for functional delivery systems [10]. They can be found in food, pharmaceuticals, cosmetics, the textile industry, conversion and fermentation processes, and environmental and other chemical systems and applications. However, the development of hosts is still ongoing, the development of processes not only simple and reproducible, but also economically profitable is fundamental so that their widespread acceptance would be guaranteed in diverse fields such as food and drug production [11].

Regarding the importance and new advances on the field of CDs and food science, some recent reviews have been focused on the latest updates of the use of CDs in food products such as nanoparticles, nanosensors, extraction enhancers, active or smart packaging, and their most widespread use as carriers and complexes formers to protect and stabilize bioactive compounds with the aim of improving the final product [12,13]. However, future research is also focused on CDs properties and their interaction with other materials (e.g., durability, stability, or dispersion), new applications (e.g., CDs immobilization for wastewater treatment) or the development of green chemistry processes [14]. They have also been used for sequester certain molecules (i.e., cholesterol), as modulators for medicinal biomaterials, as elicitors or as synergistic agents for the production of secondary metabolites in plants [15,16].

This review will be focused on the main applications of CDs in the food industry as well as in other applications, revising toxicity and legislation aspects and performing a discussion about the advantages and disadvantages of their use. Nevertheless, some applications such as nanoparticles, nanosensors, or active packaging have not been reviewed in this manuscript. Complementary information on these fields can be found along the most recent bibliography cited in this article. 

## 2. Cyclodextrins

### 2.1. Definition

Industrially, CDs are enzymatically produced with cyclodextrin glucosyltransferase from modified starch. This is an economic process, whose production is estimated in 150 tons per year [7]. The resulting product is nontoxic, characterized by not being absorbed in the upper gastrointestinal tract and being entirely processed by the colon microflora. [18].

Natural CDs are also characterized by poor solubility. At the beginning, this was a major inconvenience since it prevented the use of CDs as effective complexing agents. It was not until the end of the 1960s when it was revealed that, by means of chemical replacements at positions 2, 3, and 6-hydroxyl, the solubility was highly increased. In addition, depending on the grade of chemical replacement and the type of the substituents, the maximum concentration of CDs in aqueous mediums was improved. The maximum concentration that most chemically modified CDs can reach is 50% (*w*/*v*) in water by carrying out substitutions at the 2, 3, and 6 hydroxyl like (2-Hydroxypropyl)-β-CDs [8].

### 2.2. Mechanism

Some parameters must be considered for the study of CDs mechanism of action. The size of the cavity is essential to choose which type of CDs is more suitable to use in the interaction. The size is related to the degree of adjustment, which is a critical parameter to achieve optimal CDs incorporation. Therefore, each type of CDs will have different types of cavities. The α-CDs possess small cavities, so that they are not able to accept many molecules. The γ-CDs are larger in size compared to most molecules that can be complexed. In addition, hydrophobic CDs loads cannot efficiently interact to simplify complex formation. Therefore, it is considered that the diameter of the cavity of β-CDs is the most appropriate for molecules such as hormones, vitamins, and other compounds commonly used in tissue and cell culture applications. All these features make β-CDs the CDs of choice as complexing agent in most cases [19].

**Figure 1:**
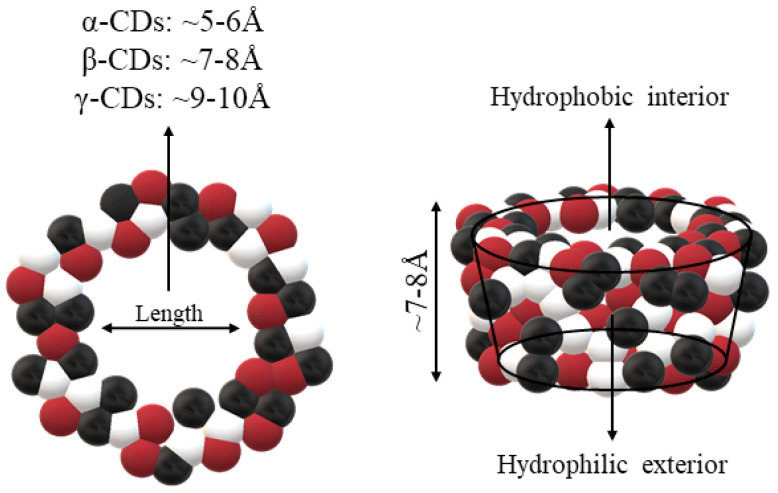
Schematic representations of cyclodextrins (CDs). Adapted from [13,21,22].

The structure of CDs allows the enclosure of hydrophobic molecules, such as vitamins and lipid soluble hormones, improving their solubility in aqueous systems. The process can be reversed by diluting the complex in a larger volume of solvent. As a result, the molecule of interest is released into the environment. The structure of the resulting complexes (Figure 1) is like a cage, with the same characteristics as those formed by cryptands, calixarenes, cyclophanes, spheres, and crown ethers. As mentioned before, all these molecules are involved in chemical reactions due to non-covalent bonds, with most of the reactions happening as the “host–guest” type [19,20].

**Figure 2:**
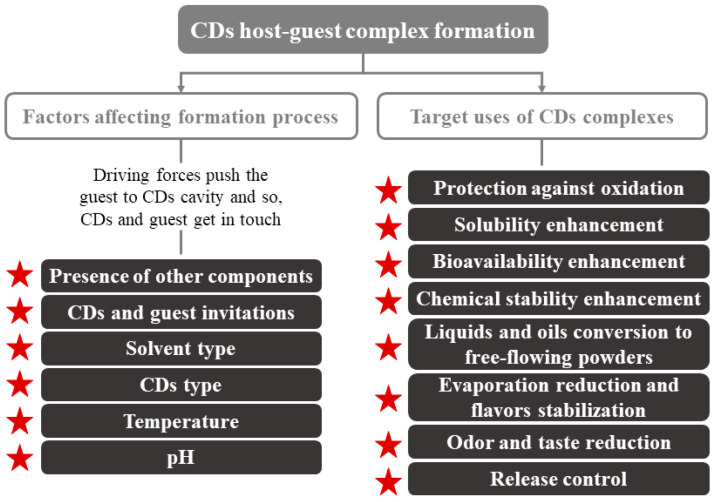
Factors affecting CDs inclusion complex formation and main application of CDs. Adapted from [22].

Regarding their physical characteristics, CDs complexes can get wet; they are almost odorless and non-hygroscopic powders [18,23]. Their mechanical properties (crystallization, flow, etc.) depend on the complex formation process. The moisture content and temperature present an enormous importance in controlling the deformation or release of the CDs-guest. Other parameters to consider can be seen in Figure 2. CDs are crystallized by two main mechanisms that, depending on the type of compounds that make up the complex (CDs and guest), turn into to two key categories of crystal packing (channel or cage structures) [5]. An example of these molecules is the flavor/β-CDs complexes, which are formed after a co-crystallization, kneading, and suspension process [18].

## 3. Applications in the Food Industry

CDs are used primarily in foods for the encapsulation of compounds of interest and the improvement of water retention, since they are hygroscopic compounds [18]. Their use can enhance several technological advantages, such as more homogeneous compositions that are easier to be standardized [18,24]. Numerous applications of CDs have been described, e.g., improving the organoleptic quality (total or partial elimination of undesired flavors/odors), increasing food shelf life, component sequestration, and pickering emulsions, among others (Figure 2). 

### 3.1. Improving Sensorial Qualities

#### 3.1.1. Color

Food color is the first quality parameter assessed by customers, so it is a key parameter of food quality [25]. CDs can be applied to modulate food color by increasing solubility and chemical stability of coloring compounds (natural ones and coloring components produced during food processing). They can provoke the inhibition of pro-browning polyphenol-oxidase reactions by complexing with several substrates or cofactors (e.g., chlorogenic acid, polyphenols, cinnamic acid, Cu^2+^) [22]. Several studies have proven the utility of CDs in food science. For instance, a study showed that the natural pigments curcumin and lycopene can form a complex with CDs, improving their solubility and reducing the degree of oxidation compared to the compounds separately [26]. Another study, conducted with chopped ginger root, showed that by adding 1–4% of CDs, the sample could be enzymatic browning stabilized for four weeks at 5 °C while being vacuum sealed. A similar inhibitory effect was observed in another study carried out with maltosyl-β-CDs in apple and pear juices. The mechanism consists of preventing ascorbic acid oxidation by an antioxidant effect, which maintains the color and the food quality [5]. α-, β-, and γ-CDs (the only CDs allowed in food industry by the U.S. Food and Drug Administration and EU) are commonly used in the elaboration of different juices to improve the color of the final product. The addition of these molecules has other effects as they can also change the concentration of individual volatile molecules as well as their chemical grouping. In the case of pear juice, adding α-CDs was recommended. By adding this molecule, the overall quality of the juice was increased since browning reactions were reduced and no significant loss of aroma quality is produced [27].

#### 3.1.2. Flavor

Flavoring substances are historic in food, although their direct use presents a series of disadvantages like having high volatility and sensitivity to light and heat. Part of these inconveniences can be solved with the CDs based encapsulation of food flavors, which is a frequent and simple solution to maintain the stability [22,24,28]. The price of the resulting β-CDs encapsulated flavor (USD $5–6 per kg) would not be much higher than other microencapsulated flavors price. CDs encapsulation provides an effective protection of each flavor component found in a multicomponent food system to whichever process it has been subjected (freezing, thawing, and/or microwaving). This factor is very important since the substances responsible for flavor usually involve numerous compounds, so it is interesting that all these molecules become part of the complex without seeing their organoleptic properties altered [18,29,30]. This method can be also used in oils to achieve a manipulability powder that can be added to food [22,24]. The liberation of aroma in these complexes can be controlled by slow guest liberation, mask off-notes of aromatic components by affinity with CDs cavity, and increase food flavor by water dissociation of aroma due to the polar external part of CDs [22]. Due to the properties of CDs-complexes, they can be applied to enhance flavor before extrusion, being a promising alternative for applying during the process [31]. As for the evolution of aromas over time, α-, β-, and γ-CDs are the best for initial flavor retention, α being better than γ for avoiding the loss of volatiles after storage [32,33]. Some examples of the application of CDs in the flavor and taste can be seen in Table 2.

#### 3.1.3. Taste

Bitterness is one of the factors that can generate the rejection of a food product. However, there are exceptions to this rule, as some products are expected to have a certain degree of bitterness, like coffee, beer, or wine [29]. By using appropriate CDs, the bitter taste of certain substances may be totally or partially eliminated since complexed compounds cannot react in the oral cavity with the taste buds. This type of taste is not perceived as only dissolved substances have flavor. This system has been applied to the bitter and astringent compounds of foods (e.g., soy), beverages (e.g., naringin in citrus juice or chlorogenic acid and polyphenols in coffee), cigarette smoke (nicotine), or oral care products or drugs [49].

The mechanism of action consists of forming complexes of enough stability with the selected CDs to make the substance, that gives the unwanted taste, insoluble in water and, therefore, in saliva, and do not cause a bad taste sensation. The effectiveness of the process will depend on the value of the complex association constant (usually 10^1^–10^4^ molK^−1^), pH (less stable complexes with ionized guest molecules), and guest/host ratio (the higher possible molar excess, the better) [49]. Finally, the complex is released throughout the digestive system. Considering this, CDs are one of the best methods for masking the unpleasant taste [22]. The most relevant publications dealing with the elimination of unwanted tastes focus on the positive effect of β-CDs, the possibilities of α-, γ-, hydroxypropyl-CDs, and maltosyl-CDs having not yet been explored. CDs can also be used in seafood and meat products, to improve texture [29]. Other cases of study of CDs for food taste improvement can be seen in Table 2.

### 3.2. Improving Shelf Life

CDs can protect several compounds present in foods from reactions such as oxidation, light induced reactions, heat promoted decomposition, self-decomposition, and loss through volatility or sublimation [24]. The encapsulation of CDs with lipophilic food ingredients, physically and chemically, improves the stability of flavors, vitamins, dyes, and unsaturated fats, among others. Consequently, the shelf life of the product will be increased [18]. As an example, an in vitro study demonstrated that CDs encapsulation improved the stability of rosemary bioactive compounds [24]. Different accelerated and long-term storage stability tests showed that ingredients complexed with CDs have a longer life than those traditionally formulated. Another study tested twelve different complexes with β-CDs stored for 14 years. The results showed that encapsulation resulted in a notable improvement of the stability during long-term storage. Its preserving power depends on factors that affect the host–guest union such as the structure, the polarity of the compounds, or their geometry. Different studies showed that the greatest protective effect is observed in flavors with terpenoid, phenylpropane, and alkylsulfide structures [18]. In a recent study, different nonalcoholic beverages used limonene complexes with α-, β-, and γ-CDs to improve flavor and shelf life. The study showed that although limonene content diminished in all cases, it did so to a lesser extent once β-CDs/limonene complexes were adjoined. After 10 days, which mimic nine months of storage, 40% of limonene complexed remained in the model drink [50].

#### 3.2.1. Against Oxidation

CDs can form complexes with ingredients (flavors, unsaturated fatty acids, dyes, etc.) sensitive to oxygen or oxidizing substances, which in most cases leads to an improvement in the stability of encapsulated substrates. Several studies have shown that complexing by means of this type of compounds almost completely prevents these oxidizable substances from undergoing chemical modifications, even when warehoused in an atmosphere of 100% oxygen [18]. Another study showed that using cinnamon-CDs complexes in the manufacture of dried apple slices with cinnamon flavor not only prevented a decrease in the concentration of this compound due to evaporation, but also protected it from oxidation [51].

#### 3.2.2. Against Light-Induced Decomposition

CDs can be also used to protect compounds of interest from deterioration factors such as light, heat, or oxidation. In addition, if the CDs cavity is filled, the entry of other molecules is prevented, so no unwanted reactions occur. Another mechanism of action is preventing reactive molecules from approaching the active sites of the host molecule. For instance, CDs have been used to protect vitamins and pharmaceutical products that contain easily oxidizable double bonds (e.g., prostaglandins). It has been demonstrated that hydroxypropyl-β-CDs protected peptides from hydrolysis and their consequent loss of ability [51].

#### 3.2.3. Against Heat-Induced Changes

Another important problematic event is thermal degradation of natural compounds. In most cases, the application of heat causes the volatilization of less stable compounds, which might have interesting biological properties. One possible solution can be the encapsulation of bioactive compounds with CDs, resulting in a complex that would provide a barrier for preventing their loss [52]. Several studies have shown that these complexes are very useful when it comes to protecting volatile flavor compounds and essential oils against heat, generally achieving better flavor retention with CDs than with various traditional formulations [53]. For example, this system has been used in vitro for the encapsulation of vitamin A palmitate to produce enriched foods using β-CDs. As a result, there is an increase of both, in its solubility in aqueous media and in its stability against different external factors (temperature, light, and oxygen) [54]. Other examples of the application of these complexes to protect several compounds can be observed in Table 3.

### 3.3. Modifying Solubility

**Figure 3:**
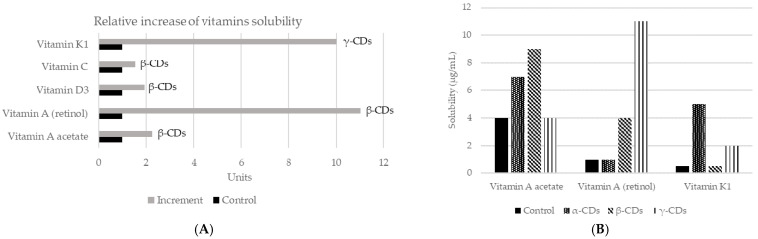
Changes in solubility in water of different vitamins according to the use of different CDs. (**A**) Relative increase of the solubility of vitamins using different CDs complexes. Adapted from [74,75]. (**B**) Vitamins solubility (µg/mL) when the three main types of CDs applied in a concentration of 5%, using saline solution as control and measured by mass spectrometry [74]. More recently, new studies have assessed the solubility of vitamin E + large ring cyclodextrins [76] and vitamin A together with β-CDs [77].

As mentioned before, CDs are capable of changing the solubility of a compound [67]. They have the ability to form stable emulsions of water in oil (e.g., mayonnaise, salad dressings), due to differences in polarity between the inside and outside of the molecule [18]. In addition, CDs can also increase the solubility of certain compounds in water, by forming dynamic, noncovalent, water-soluble inclusion complexes [68]. However, in many cases, the solubility of the complex is not appropriate, so it is necessary to modify the external surface of CDs. Neutral (hydroxypropyl) or ionic groups (hydroxy, carboxymethyl, tertiary amine, or quaternary amine) can be used to increase the solubility up to 60%. On the other hand, to improve solubility in organic solvents, the modification is carried out with aliphatic groups or smaller groups (hexyl, acetyl). Thus, complexation with CDs is a mechanism to increase or decrease the solubility of a guest component. A common example of its application is to reduce the bitterness in citrus juices, by creating naringin-β-CDs complex. In fact, the rate of transformation of naringin to naringenin in inclusion complexes or free can reach 98.7% and 56.2%, respectively. It might be concluded that β-CDs can improve the aqueous solubility, which also means that the rate of enzymatic hydrolysis of naringin will be increased [69]. The increase in solubility will also affect aromatic compounds, such as vanillin, used at the vanillin/β-CDs inclusion complex. This complex increased solubility in water with respect to the free compound. Moreover, the formation of this complex protects vanillin inside the CDs cavity, which avoids damage from several factors such as oxidation according to the results obtained in differential scanning calorimetry (DSC) studies. Therefore, the vanillin/β-CDs complex can be used as a food additive for its higher antioxidant activity [70]. CDs may be also applied to improve the solubility of vitamins (Figure 3). The union of β-CDs with the essential oil from guava leaves also increases solubility and stability. The resulting complex presented antioxidant (against light) and antibacterial (*Staphylococcus aureus* and *Escherichia coli*) activities [71]. Similar effects were achieved by encapsulation of black pepper essential oil or yarrow essential oil [72,73].

### 3.4. Sequestration of Selected Components

Among the most recent CDs applications, reduction of unwanted compounds (flavor, trans-fats, allergens, toxins) is included [24]. Allergens can be avoided with β-CDs. It has been demonstrated that they form host–guest systems with allergenic aroma molecules (eugenol, isoeugenol, benzyl alcohol, or anisyl alcohol) and proteins [78,79]. An example of this possible application is the preparation of soybean milk of low allergenicity. They are also able of forming complexes with mycotoxins like ochratoxin A from cereals, coffee, beer, wine, and cocoa [80]. CDs have been demonstrated to sequestrate other mycotoxins, such as aflatoxin, ochratoxin, patulin, zearalenone, zearalenol, and citrinin [79]. A study used 1% β-CDs during apple juice processing to reduce mycotoxins (patulin) and inhibit enzymatic browning by 70% and 75%, respectively [81].

Other application of CDs is the sequestration of cholesterol from food products [24,82]. They can be applied in food with high content of these fatty acids to make it healthier. This is the case of milk, butter, and egg yolks [83]. In fact, this property is widely used in the industry to produce products without cholesterol since this compound is retained in the β-CDs cavity. CDs did also sequester reducing sugars capable of reacting with proteins (Maillard reaction) and that could give an undesirable color and adverse effects in the nervous system and fertility, being a possible carcinogen. The mechanism is based on the complexation of proteins with CDs, which protect them from reaction. They can also be applied to decrease acrylamide content in food products and food intermediates [84].

### 3.5. Pickering Emulsions

Pickering emulsions are strictly defined as emulsions that are stabilized by an adsorbed layer of solid particles at the emulsion drop surface [85]. Their properties are usually determined by particle size, particle wettability, particle concentration, oil/water ratio, pH, salt concentration, and solvent type [86]. CDs can form CDs-oil complexes, however, in most cases, high concentration of CDs is needed. To solve this inconvenience, modified CDs can be used such as soft colloidal CDs polymer (CDs nanogel) [87]. Thermal stability in water of this type of emulsions with different oils has been investigated, observing activity at room temperature and the dissolution/fusion of inclusion complexes with high melting temperatures (near to or higher than 100 °C) [86]. Another study has considered the union between β-CDs and octadecenylsuccinic anhydride (ODS) under alkaline conditions. ODS-β-CDs particles exhibited a higher emulsifying capacity compared to β-CDs. The resulting pickering emulsions formed by ODS-β-CDs particles were more stable during storage [88].

### 3.6. Other Food Applications

Apart from food processing, CDs also have other technological advantages such as improving nutritional properties and for developing nutraceutical products [18]. Several studies have been conducted in this aspect. α-CDs complex can be used to keep certain products (cereal, snacks) crunchy after storage and also as soluble dietary fiber in beverages and foods [30]. Other CDs complexes can be used in the preservation of food. They can be used indirectly in the prevention of microbial growth, being added in plastic packaging films. In this way, they preserve food during storage and also prevent loss of aroma [18]. Finally, CDs have been demonstrated to be useful to remove contaminants from food products, including herbicides, insecticides, fungicides, repellents, pheromones, and growth regulators [5].

## 4. Other Applications

### 4.1. Pharmaceutical Applications

CDs are used for numerous purposes in pharmaceutical applications (Table 4). In drug formulation, CDs have been described to increase the bioavailability, solubility, stability, reduce hemolysis and adverse effects, prevent admixture incompatibilities and act as excipients, among other uses [89,90,91] (Table 5). Improving the solubility of drugs is interesting, since the compound will have greater therapeutic efficacy, and lower doses will be necessary [68]. Numerous anticancer CDs-based drugs are being clinically evaluated [91]. CDs have been also used to delivery oligosaccharides, proteins, and oligonucleotides due to their capacity to interact with cellular membranes, improving cellular uptake. Another application is the delivery of gene-therapeutic agents, such as plasmids, viral vectors, and antisense constructs [92]. CDs have been demonstrated to protect carbamates in vitro, increasing their half-life [93]. Its ability to sequestrate determined compounds is also useful for pharmaceutical applications. CDs can sequester neuroactive steroids, which are potent modulators of GABA_A_ receptors [94]. They can be used to elaborate pickering emulsions that can be used for topical applications in the formulation of antifungal econazole derivatives delivery [95].

### 4.2. Cosmetics and Personal Care

CDs are also used is cosmetics [96]. Its application has numerous advantages, such as stabilizing compounds, obtaining odors and flavors of greater acceptability, improving the action of the compound by transforming a liquid constituent to a solid form, reducing vapor pressure, changing the solubility in water, and improving the thermal stability of oils, among others [6]. They are used in the suppression of the volatility of perfumes, air fresheners, and detergents since they allow a controlled release of fragrances from the host–guest complex, producing more doubtful fragrances [92]. They are also used in the formulation of toothpaste, skin creams, fabric softeners (liquid and solid), paper towels, tissues, and underarm shields. Therefore, CDs represent a valid formulation support, since they can improve the performance of the resulting product and solving problems that may arise during its formulation. Several studies have been performed to study different cosmetical applications of CDs. For instance, an in vitro study has proven that CDs are a useful delivery vehicle of ferulic acid (a compound with well-known antioxidant and photoprotective properties), improving its photo-stability, which could be an interesting property for cosmetic formulations [102].

### 4.3. Packing and Textile Industry

In recent years, the textile industry has directed its research towards making functional and sustainable fabrics [89]. In this field, the β-CDs can play a fundamental role since it can form complexes with different types of compounds, which makes a new wide variety of textile products and applications with advanced properties, such as antimicrobial or photoprotective. The incorporation of CDs to the textiles may also serve to deliver aromas and capture malodors (sweat, smoke) or increase the ability of fabrics to retain dyes with the consequential benefit of decreasing the amount lost in wastewater [89,92,103]. Moreover, they can also be flame-retardants [103]. In medicine, medical tissues containing CDs are used to release chemical compounds (both topically or inside the body) with beneficial properties, such as antibacterial, anti-allergic, antifungal, anti-inflammatory, and protection against insects [23]. Different studies seem to indicate that the most promising way to bond CDs in fabrics would be to generate complexes of monochlorotriazinyl-CDs by binding CDs with trichlorotriazines [104]. β-CDs can also be used as a novel molecular phosphorescent material as light sensitive phosphorescent color changes have been detected, which makes it a promising candidate as dynamically photo-functional material [105]. Other applications of CDs in fabrics and textiles can be seen in Table 6.

### 4.4. Bioconversion and Fermentation

Bioconversion and fermentation processes are frequently limited due to the toxic or inflammatory effect produced by the substrate or product in the catalyst. In addition, the medium is also of great importance given that most of the organic substrates are lipophilic and, thus, they have low water solubility and the catalyst is usually more active. As a result, only a small part of the substrate is reachable to the biocatalyst [92]. Different techniques have been carried out to overcome these problems. Among them, there are the addition of the inhibitory substrate to the fed batch, in situ recovery of the inhibitory product or the solution of the lipophilic substrate with surfactants and organic solvents. The solution may be achieved by the use of CDs [120]. Some studies have used CDs to improve the production efficiency of different compounds. For instance, production efficiency of spiramycin was enhanced by CDs [121]. Modified β-CDs also increase the rate of deacetylation of spironolactone [122].

### 4.5. Environment

The role of this type of compounds in the environmental field is mainly due to its ability to solubilize organic pollutants, enhance and eliminate organic contaminants and heavy metals from the environment. In this field, CDs studies and new applications are expected to grow in the following years. β-CDs have been used in the adsorption of contaminants since they do not generate additional pollution [123]. Regarding water treatment, several possible applications have been described. An example of this application would be the treatment of textile wastewater contaminated with dyes [103]. CDs nanosponge adsorbents have been studied for water treatment. To achieve this, CDs nanosponges are modified with adsorbent nanomaterials (nanotubes made with carbon, titanium oxide, and silver nanoparticles). The obtained results proved the efficiency of these systems for removing contaminants from water [124].

### 4.6. Catalytic

In recent years, research efforts have been focused on the development of CDs capable of catalyzing organic reactions since they can bind to the substrate and form inclusion complexes with small compounds. CDs have been considered as artificial enzymes, generally characterized by having substrate specificity due to the structure and properties of the CDs cavity, even showing stereospecificity [125]. These complexes have been used for advanced homogeneous or heterogeneous catalytic processes. Moreover, they can be used to make alternative reaction media, such as hydrogels or low melting mixtures. These mixtures are capable of stabilizing active catalytic species, resulting in an increase of the catalytic activities and selectivity in transition metal reactions. In addition, after the catalysis is processed, artificial enzymes can be recovered by only a phase separation. With this catalytic systems process, safety is greatly improved [126]. Some advantages of CDs-based molecular catalysts are their easy preparation and isolation, economical and effortlessly obtainable starting materials, possibility of reuse and likelihood of acting in mild aqueous conditions (ecologically sustainable technology) [127].

A recent advance in this field consists of using CDs as an enzymatic mimic, since a molecular recognition phenomenon occurs due to the groups substituted in the CDs [92]. For example, β- or γ-CDs can be used in benzoin condensation with a rate increase of 7-fold [128]. Another example is a molecular catalyst of Pd/β-CDs, which shapes inclusion complexes with small organic compounds. It was utilized with remarkable results to reduce toxic aromatic components and the degradation of harmful dyes [129].

### 4.7. Analytical

CDs and their derivatives are utilized in a wide diversity of analytical chemistry fields, especially in analytical separations as they could distinguish between positional isomers, functional groups, homologues, and enantiomers [92]. They can be used in different areas such as chromatography, waste-water treatment, and other separation techniques (extraction, complex formation) [130]. For instance, they are used in chromatographic separations as they increase the selectivity in comparison to separations carried out with an eluent and stationary phase without any additional help [131]. Nevertheless, further development is necessary to reduce preparation costs, particularly for environmental applications. CDs could be also used as a reagent in several methods, including UV-visible spectrophotometry, photoluminescence and nuclear magnetic resonance [132]. This use is related to the ability of CDs to increase the emission intensity of the reactions that are taking place due to several aspects. Higher reaction rates and a greater efficiency in the process of excitation and protection of species that emit the quenching phenomena are some examples of these aspects [132].

## 5. Advantages and Disadvantages of Their Use

Using CDs complexes in food systems has several advantages, which have been explained relating them to their application in the industry. These advantages can be grouped in blocks. The first of these would be the increase of shelf life of the resulting products, due to the ability of CDs to protect compounds against different factors such as oxidation, light-induced reactions, decomposition, and thermal decomposition. Therefore, these types of molecules act as a potential stabilizing agent. For example, making possible its application to target orally administered water-insoluble drugs. They can also increase the stability of emulsions. The second block is related to physical properties changes, which has advantages such as increasing the solubility of different compounds. They also eliminate hygroscopicity, which is the ability to absorb moisture from the surrounding environment. Rheological properties as well as viscosity are also modified by the physical characteristics of these compounds. Other advantage is that its structure allows the union with a great variety of compounds, achieving even high order complexes. Moreover, as CDs have a well-defined chemical structure, they possess many potential sites for chemical modification or conjugation, giving rise to a great variety of complexes. In terms of their advantages at technological level, CDs are characterized for being stable, having simple dosing and being able to handle dry powders. This makes the reduction of packing and storage costs possible, resulting in more economical technological processes and manpower saving. Standardization allows products that do not vary over time to enter in the market, which means that they are more accepted by consumers. The ability to sequester unwanted compounds is also a big factor in terms of flavor. This quality is closely related to its application when it comes to improve sensory quality since CDs are able to eliminate or reduce undesired tastes and odors and preserve the desirable ones protecting them from loss by evaporation and sublimation. They can also form complexes that allow the development of new additives in order to improve important factors in the food industry such as color and flavor. In addition, CDs would satisfy consumers’ demand for more natural products, being one of the most promising compounds to use as additives to improve sensory quality.

Despite everything, they also present a series of drawbacks. Even though they are characterized for having a low toxicity, in some cases, it has been shown that they can have an irritating effect. Thus, there are safety concerns, which limit their use for parenteral administration showing renal toxicity in most species. However, despite these adverse effects, their toxicity as well as its immunogenicity remains low, which is the reason that CDs have extremely appealing pharmaceutical applications. In many cases, they can accelerate reactions, for example, the hydrolysis of esters, amides and organophosphates, decarboxylation and oxidation reactions. Furthermore, royalty payments may be required as many patents are still enforceable. In all cases, binding constants of the complex need to be optimum, especially in drugs for pharmacokinetics.

## 6. Toxicity and Legislation

When CDs were discovered, they were considered poisonous substances, so their application in complex formation was considered an anecdote. Afterwards, it was shown that they had no toxic effects and that they could also be very versatile for their protective properties [29]. β-CDs have been the most studied within the CDs, having proved its safety. At the maximum dose of β-CDs orally presented, no mortality has been observed. As a result, a nontoxic quantity of 650 mg/Kg/day (1.25% of diet) of β-CDs was designated in rats. In contrast, the injection of β-CDs resulted in the decease of the animals, as it caused kidney damage due to crystallization of the complex in the kidneys [51]. It is worth mentioning that only very little β-CDs were completely absorbed as salivary or pancreatic amylases had no effect. Therefore, the complex reached the colon without having undergone any modification and enzymes from the intestinal flora opened CDs ring generating a maltoheptaose. This compound is catabolized like the other starch fragments that reached the colon [51]. CDs are widely used in food since its applications include acting as a vehicle of ingredients (flavor, vitamins, polyunsaturated fatty acids, etc.) and as a stabilizer. The available data estimates an acceptable daily intake (ADI) for all its applications in foods of 4.1 g/person/day for consumers of foods containing γ-CDs. Several studies have been conducted to verify the risks of this consumption [133]. Different studies concluded that γ-CDs had no toxic effects and were well tolerated by the body. This may be because their similar metabolism to starch and linear dextrin, according to the results obtained in metabolic studies in rats (rapidly and digested by amylases present in saliva and pancreatic juice). Moreover, γ-CDs will not affect the absorption of lipophilic nutrients since inclusion complexes are reversible and they have a similar gastrointestinal tolerance of maltodextrin. All these studies included γ-CDs as a product generally recognized as safe (GRAS) for its use in food [134]. The acute oral LD_50_ was 8000 mg/kg for rats [51]. It should also be added that CDs, as well as their hydrophilic derivatives, can only penetrate lipophilic membranes with considerable difficulty. Consequently, its absorption in the gastrointestinal tract is very low, thus, its oral administration presents almost no toxicity [103]. The World Health Organization (WHO) concluded that, according to all available studies on α-, β-, and γ-CDs and their allocated ADIs, these food components contained an almost insignificant toxicity. Therefore, according to the available information, their consumption levels (total dietary intake) and their acceptable background levels in food, CDs do not entail a threat to health. Not showing cytotoxic effects is vitally important in applications such as food and flavors but also in cosmetics, packaging, textiles, separation processes, environmental protection, fermentation, and catalysis [5]. These types of compounds have not shown any allergic impact according to OECD experiments. Since 2000, β-CDs are commercialized in Germany as a food additive [103].

Many studies concerning the toxicity of CDs are based on their medical applications. Even though the safety aspects should have been considered during the development, and safety assessment of each medication should be clearly established on the data sheet, in practice, it does not occur. However, a high dose of CDs has adverse effects. Although its oral availability is very low, high doses can lead to reversible diarrhea and caecum enlargement. In addition, depending on the dose, the permeability of the tissues and, consequently, the bioavailability of the active substances administered, may also be altered. In cases of high systematic exposures in animals, nephrotoxic effects have been observed. So far, there is no evidence of these effects in humans [135,136,137]. Moreover, as there is not enough information about the effects of CDs in children under two years old, a case-by-case judgment regarding the risk/benefit to the patient must be performed. Nevertheless, CDs are not currently included in the European Commission Guide of excipients on the label and the package leaflet of medicinal products for anthropological use [138]. For example, the toxicity of hydroxypropyl-β-CDs (HP-β-CDs) was studied, i.e., a substance that has been extensively used as a solubilizing agent in the pharmaceutical industry for many years. Despite this, no studies on solubilizing capacity and toxicity have been conducted according to the degree of substitution. The obtained results showed that the best option for both solubility and toxicity would be HP-β-CDs with low degree of substitution (DS). However, further studies are still necessary since the comparison of HP-β-CDs toxicity with diverse DS must be executed in humans considering its dependence on the species. 

The current legislation regarding CDs varies between Europe and other countries. Within Europe, as they are considered as food additives, they are subjected to the same legislation. In the USA α-, β-, and γ-CDs are considered a GRAS food additive. However, in Japan the three CDs are recognized as natural products. Therefore, their commercialization in the food sector will be only restricted by considerations of purity. Other countries in which α- and γ-CDs are considered food (novel foods) are Australia and New Zealand since more than fifteen years ago [30]. Despite α-, β-, and γ- CDs are being used as food additives in those countries, in Europe β-CDs has been approved as an additive (E-459) and lately α-CDs as a novel food. γ-CDs can be also used in the food industry; however, its use is restricted to those countries in which it has been approved. An outline of the state of the legislation worldwide can be seen in Table 7.

According to different reports (1996) from the Scientific Committee on Food (SCF), the ADI of β-CDs (E 459) is 5 mg/kg of body weight per day. Giving the available reported use and use levels, the EFSA Panel also determined that the ADI was surpassed in the refined brand-loyal scenario (contemplated as the most relevant scenario) on average in whole population groups excluding babies and in all population groups in the 95th percentile [137]. According to data, FAO/WHO Expert Committee on Food Additives (JECFA) proposed a maximum level of β-CDs in foods of 5 mg/kg per day. Owing to their positive toxicological profiles, neither α- nor γ-CDs have a defined ADI. Furthermore, in July 2005 the U.S. Environmental Protection Agency (EPA) eliminated the need to create a maximum allowed level for residues of α-, β- and γ-CDs on several food supplies. Regarding the assessment of the levels of exposure on animal and human studies and associated adverse effects, it is considered that α-CDs have very low toxicity and that, at the proposed levels of dietary exposure when used as a food ingredient, would not have toxicological effects.

## 7. Future Perspectives and Conclusions

CDs and their derivatives have an extensive diversity of uses in various fields (food, cosmetics, and drugs), but especially on the food industry and, thus, their use have increased in recent years. These applications are mainly due to the capacity they possess to form host–guest complexes with a wide diversity of compounds. This type of molecular encapsulation improves the stability of flavors, vitamins, colorants, unsaturated fats, and other lipophilic molecules in physical and chemical senses leading to extended product shelf life. Furthermore, by using this technology, sensorial qualities can be improved, and microbiological contaminations can be avoided. In addition, due to their low toxicity, they can be applied without risk to human health, not only resulting in healthier and more functional products, but also less perishable. Cases of sold CDs-based food products for demonstration of the importance of CDs technology in the food industry were reviewed.

CDs are an exceptional type of building blocks in innovative molecular architecture due to their low toxicity, their capacity to hold, orient, conceal, modify their chirality, and isolate their guest compounds. All these properties make possible to use them not only as excipients, but also as extenders, chelating agents, or other multipurpose technological tools. Another application of great interest is the sequestration of toxic compounds, which makes them capable of modifying the toxicity of the substrate [24]. Their use in food, agriculture, pharmaceutical products, and chromatographic techniques has increased considerably in the last decade. Therefore, the unique architecture of CDs makes them a significant option in drug advance, in chiral separations and as complexing agents in food, cosmetics, and pharmaceutical manufactures. Among them, it is worth mentioning the scope of food since consumers’ demand more natural and healthy products with the consequent expansion of the market of functional foods and nutraceutical products. This can be achieved with the application of CDs, which has a promising future.

Another emerging use of CDs is as encapsulation agents at molecular level due to their ability to absorb whole molecules or part of them into their cavity. They can be the key to many future encapsulated formulation solutions. It is also expected that they will continue to be applied due to many other advantages, including increased solubility, stability against light, heat and oxidizing conditions, and decreased volatility. It must also be considered that not only pure CDs but also their derivatives can be used, which increases their possible applications. However, to carry out all these processes, a few main concerns are cost reduction and the efficiency of manufacturing. In recent years, there have been great advances in this regard, which makes it likely to increase their applications. In addition, several studies showed that the formulation of CDs derivatives have better stability than those of the traditionally formulated ones. The number of publications carried out in recent decades is a clear example of the growing interest in the potential applications of CDs.

## Figures and Tables

**Table 1:** Main cyclodextrins (CDs) properties [5,17].

Properties	Unit	α-CDs	β-CDs	γ-CDs
Formula		C_36_H_60_O_30_	C_42_H_70_O_35_	C_48_H_80_O_40_
Structure		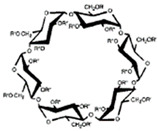	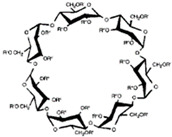	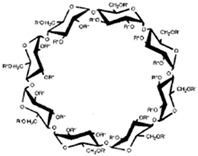
Mol wt		972.84	1134.98	1297.12
Glucopyranose units		6	7	8
Solubility (water, 25 °C)	% *w*/*v*	14.5	1.85	23.2
Outer diameter	Å	14.6	15.4	17.5
Cavity diameter	Å	4.7–5.3	6.0–6.5	7.5–8.3
Height of torus	Å	7.9	7.9	7.9
Cavity volume	Å^3^	174	262	427
Crystal form		Hexagonal plates	Monoclinic parallelograms	Quadratic prisms
Others			Most accessible, lowest-priced and generally the most useful	

**Table 2:** Studies of cyclodextrins (CDs) for food flavor and taste improvement.

CDs	Extract	Compounds	Characteristic	Effect	Ref.
**A. Food Flavor Improvement**					
α	Shiitake	Cyclic sulphur compounds	Shiitake mushroom	Flavor retention	[34]
α	European pear	Five ester types	Pear	Heat protection at 120 °C for 60 min.	[35]
β	Food	-	-	Encapsulation the best protection against heat and evaporation	[36,37]
β	Food	Several volatile compounds	-	Protection during high temperature short time extrusion cooking process	[38]
β	Polysaccharide solutions	ketones, hexanal, t–2-hexenal, ethyl butanoate and 1-hexanol	-	Retain some aroma compounds during thermal processes (cooking, pasteurization)	[39]
β	Corn starch	Eugenol	Clove	79% odor retention during extrusion	[40]
β	Goat milk and its yogurt	4-methyloctanoic acid	Goat flavor	Reduce goat flavor	[41]
β	Thermally processed foods	l-menthol	Menthol	Improved flavor retention	[42]
α, β, γ	Aqueous ethanol	l-menthol, ethyl butyrate, ethyl hexanoate, benzaldehyde, citral, and methyl anthranilate	-	Temperature dependent	[28]
**B. Food taste improvement**					
α	Soy protein	Phenylalanine, tryptophane, tyrosine, isoleucine, proline and histidine	Taste modification	Reduce bitter taste	[43]
β	Milk casein hydrolysate	-	Bitter	Bitter taste eliminated by adding 10% β-CDs to the protein hydrolysate	[29]
β	β-polymers	Limonin, naringin	Bitter	Debittering agents	[44]
β	Canned citrus and citrus juice	Naringin, limonin, hesperidin	Bitter, precipitation	Reduce bitter taste of naringin, limonin, and hesperidin and prevent precipitation	[22]
β	Fish oil	-	Taste, oxidation	Eliminate unpleasant taste, smell, and stabilization against oxidation	[45]
γ	Ginseng	-	Bitter	Debittering agent	[46]
α, β	Navel orange and grapefruit juices	Limonin, naringin	Bitter	Improve flavor	[47]
β, γ	Caffeine and bitter natural extracts (artichoke leaves, aloe, and gentian)	β- and γ-CDs linked to chitosan through succinyl or maleyl bridges	Bitter	Bitter-masking properties	[48]

**Table 3:** Studies of cyclodextrins (CDs) for heat protection of food ingredients.

CDs	Subtract	Properties	Study	Effect	Info	Ref.
β	2-nonanone	Aromatic, antifungal	TGA, DSC, against *B. cinereal*	Improve antifungal, thermal stability	complex 1:0.5 (80% growth inhibition).	[55]
β	cyanidin-3-O-glucoside	Several	DSC	Improve bioavailability, thermal protection	-	[56]
β	*S. baicalensis* BA	Anti-inflammatory, antioxidant, antitumor		Increase solubility, stability	13672.67 L/mol	[57]
β	*S. salar* EO		DSC, KFT	Thermal and oxidative stability	Complex 1:1 and 3:1	[58]
β	Benzyl isothiocyanate (papaya)	Antimicrobial	DSC, TGA	Improve stability, controlled release	600.8 L/mol	[59]
β	*O. basilicum* EO	Aromatic, medicinal	GC-MS	Improve stability against air/oxygen and temperature	-	[60]
β	Methanolic extract of *H. perforatum*	Antioxidant	DSC	Intact at temperatures at which the free extract was oxidized	Food supplement or a novel additive to enhance the antioxidant capacity of fresh or thermally processed food	[61]
β	Garlic	Antimicrobial, antioxidant	TGA, DSC, SEM	Thermal and oxidative stability	Nanoencapsulation yields >60%	[62]
β	Garlic oil	Antimicrobial, antioxidant	DSC	Improve protection against oxidation	Complex 1:1	[63]
β	Oils	Antimicrobial	DSC, against *S. enterica* and *L. innocua*	Thermal protection	Masking the sensory effect of the attributes of antimicrobial agents and potentiate their activity.	[64]
HP	Clove EO	Antioxidant	DPPH	Prevent degradation and loss of active compounds. prolong shelf life	Complex 1:1	[65]
γ	Geraniol	Aromatic, to treat infectious diseases, preserve food	SEM analysis	High thermal stability and enhanced durability of active agents and functional food ingredients	-	[66]

HP: hydroxypropyl beta-cyclodextrin; BA: baicalein; EO: essential oil, DSC: differential scanning calorimetry, TGA: thermogravimetric analysis, GC-MS: gas chromatography-mass spectrometry, DPPH: 2,2-diphenyl-1-picryl-hydrazyl-hydrate, SEM: scanning electron microscope, KFT: Karl Fischer titration.

**Table 4:** Examples of some cyclodextrins (CDs) studied in pharmaceutical applications. Adapted from [87,96,97,98,99,100].

CDs	Drug	Trade Name	Admin. Route	Use	Market
α	Alprostadil	Prostavastin, Caverject, Edex	Intravenous	Erectile dysfunction; certain heart, lung, and blood vessel problems in infants; temporarily keep the arteriosus duct open before having a surgery	EU, Japan, USA
	Cefotiam hexetil HCl	Pansporin T	Oral	Infections	Japan
	Limaprost	Opalmon, Prorenal	Oral	Vasodilator	
β	Benexate	Ulgut, Lonmiel	Oral	Treatment of peptic ulcer	Japan
	Albendazole	Zentel, Colidetol	Oral	Anti-microbial	EU
	Gliclazide	Diamicron	Oral	Anti-diabetic	EU
	Danazol	Danatrol	Oral	Endometriosis	EU
	Dexamethasone	Glymesason	Dermal	Anti-inflammatory, treat eczema/dermatitis	Japan
	Ibuproxam	Calmatel, Deflogon	Oral, topical	Anti-inflammatory	EU
	Iodine	Mena-Gargle	Topical	Infections	Japan
	Fenoprofen	Nalfon, Mylan, Naprofen	Oral	Anti-inflammatory	EU
	Chlordiazepoxide	Transilium	Oral	Reduces anxiety	Argentina
	Isradipine	Almodipino	Oral	Enhance solubility and photostability	-
	Cephalosporin	Meiact	Oral	Antibiotic	Japan
	Nicotine	Nicorette	Sublingual	Aid to smoking cessation	EU
	Nimesulide	Nimedex, Mesulid	Oral	Analgesic, antipyretic, and anti-inflammatory	EU
	Diphenhydramin	Stada-Travel	Oral	Neurological treatments	EU
	Glimepiride	Amaryl, glimepiride ALTER, glimepiride, Roname, Tandemacte	Oral	Increase dissolution rate, time of action and efficacy	-
	Sulindac	Clinoril	Oral	Anti-inflammatory	EU
	Nitroglycerin	Nitropen	Sublingual	Treat / prevent chest pain or pressure	Japan
	Omeprazole	Omebeta	Oral	Intestinal / esophagus ulcers, reflux disease, heartburn, syndromes of stomach acid	EU
	Dinoprostone	Prostarmon E	Sublingual	Oxytocic	Japan
	Piroxicam	Brexin	Oral	Analgesic, antipyretic, anti-inflammatory	EU
	Tiaprofenic acid	Surgamyl	Oral	Analgesic, antipyretic, anti-inflammatory	EU
2-HP-β	Cisapride	Propulsid	Rectal	Gastro-esophageal reflux	EU
	Voriconazole	Voriconazole Teva, Vfenf	Oral. injection	Enhance solubility, dissolution rate, and chemical stability	EU
	Hydrocortisone	Dexocort	Buccal	Relieve the soreness of mouth ulcers and speed up healing	EU
	Rhein	Rhein	Oral	Improvement in photostability	-
	Indomethacin	Indocid	Eye drops	Anti-inflammatory	EU
	Itraconazole	Sporanox	Oral, intravenous	Fungal infections	EU, USA
	Mitomycin	Mitozytrex	Intravenous	Cancer	USA
ME-β	17β-Oestradiol	Aerodiol	Nasal spray	Menopausal climacteric symptoms	EU
	Chloramphenicol	Clorocil	Eye drops	Ear infections	EU
SP-β	Voriconazole	Vfend	Intravenous	Fungal infections	EU, USA
	Ziprasidone maleate	Geodon, Zeldox	Intramuscular	Acute agitation in adults with schizophrenia	EU, USA
2-HP-γ	Diclofenac sodium	Voltaren	Eye drops	Eye surgery, hay fever	EU

Admin route: administration route; HP: hydroxyprpyl; ME: methylated; SP: sulphobutylether; EU: Europe.

**Table 5:** Example of some improved drug functions achieved by CDs complexation. Adapted from [89,101]

Improved Function	Mechanism	Type	Drugs
Increase in bioavailability	Increased solubility and stability	β, γ, natural	Thalidomide, nimuselide, prednisolone, oteprednol etabonate, tacrolimus, sulfhamethazole
Increased availability	Increase in solid stability	β	Quinapril
Increased solubility	Forming inclusion complexes with their nonpolar molecules or functional groups	β	Bromazepan, ibuprofen, naproxen, ofloxacin, ketoralac, nimesulide, omeprazole, tenoxicam
Increased stability	Obstruction of the reactants to diffuse into the cavity and react with the protected guest	β	Metoprolol, nifedipine, quinapril
Increased absorption	Oral delivery	Β, HP-β	Ketoconazole, testosterone
	Rectal delivery	2HP-β	Flurbiprofen, carmafur, biphenyl acetic acid
	Nasal delivery	2HP-β	Morphine, antiviral drug, insulin
	Trans-dermal delivery	6-O-(carboxymethyl)-O-ethylβ	Prostaglandin
	Ocular delivery	2HP-β, β	Dexamethasone, carbonicanhydrase inhibitors
Delivery	Protein and peptide delivery	Modified CDs	Growth hormone, interleukin-2, aspartame, albumin and MABs
Reduction of local irritancy and toxicity	Forming inclusion complexes with toxicity or irritant compounds	2-HP-β (2,6-diOmethyl), β	Pilocarpine, phenothiazine euroleptics, all-transretenoic acid
Prevention of incompatibility	Prevent drug-drug or drug-additive interaction.	β, γ	Piroxicam, omeprazole

**Table 6:** Examples of studies using cyclodextrins (CDs) system on textile surface.

Activity	CDs	Compounds	Fabric	Study	Ref.
Antibacterial	MCT-β	Ag NPs, triclosan	Wool	Activity more than 75%, even after 15 washing cycles.	[106]
	MCT-β	Aqueous/alcoholic extracts from plants	Cotton	Other effects associated	[107]
	MCT-β	Miconazole nitrate	Cotton	*C. albicans*, *Aurococcus* and *Bacillus*	[108]
	MCT-β	Ag NPs	Cotton	*Staphylococcus aureus*, *E. coli*	[109]
	TCA-β	Octenidine dihydrochloride	Cotton	Reasonable activity after 20 washing cycles	[110]
	CTR	Silver (I)	Cotton	*E. coli*	[111]
	MCT-β	Ferulic acid, caffeic acid, ethyl ferulate allantoin	Hemp	Sanogenetic properties of the hemp fibers are significantly modified by the chemical treatments	[112]
Antiallergic, anti-psoriasis	2,6-di-O-methyl	Tacrolimus	Cotton	Drug delivery	[113]
Anti-psoriasis	-	Dithranol	Cotton	Clinical test	[114]
Chronic venous insufficiency	β	Troxerutin	Pa-66/PU in stockings	In vivo tests on Wistar rats, clinical studies	[115]
Anti-inflammatory, antioxidant, antitumor	β	Curcumin	Nanofibre	Two sequential stages for drug release	[116]
Against mosquitos	β	Cypermethrin, prallethrin, permethrin, N,N-diethyl-m-toluamide	Cotton	Treated fabrics retain high number of insecticides	[117,118]
	MCT-β	Limonene	Cotton	Effect of washing and storing	[119]

**Table 7:** Summary of existing legislation.

CDs	Food			Pharmacopoeia Monographs		
	US	Europe	Japan	US	Europe	Japan
α	GRAS	Novel food	Natural product	Yes	Yes	Yes
β	GRAS	Food additive	Natural product	Yes	Yes	Yes
γ	GRAS	Pending	Natural product	In progress	In progress	Yes
HP-β	-	-	-	Yes	Yes	-
